# A rare case of a giant placental chorioangioma with favorable outcome

**DOI:** 10.11604/pamj.2020.36.214.21635

**Published:** 2020-07-24

**Authors:** Konstantinos Zacharis, Stavros Kravvaritis, Theodoros Charitos, Eleni Chrysafopoulou, Anastasia Fouka

**Affiliations:** 1 Department of Obstetrics and Gynaecology, General Hospital of Lamia, Lamia, 35131, Greece

**Keywords:** Placenta, chorioangioma, non-trophoblastic tumor, prenatal diagnosis

## Abstract

Chorioangioma is the most common type of placental tumor. A primigravida woman was noted on routine scan at 21 weeks of gestation to have a placental mass measuring 1.8 x 2.2cm. A detailed ultrasound scan revealed a well circumscribed, hypoechoic lesion protruding into the amniotic cavity; hence a preliminary diagnosis of placental chorioangioma was made and close prenatal surveillance was scheduled. At 34 weeks of gestation, the mass was measuring 6.27 x 5.38cm. The patient experienced reduced fetal movements at 37 weeks gestation. A small-for-gestational age but normal female neonate was delivered by caesarean section. Histopathological analysis of the placenta confirmed the diagnosis. According to our case, a giant placental chorioangioma may have a favorable outcome without any medical intervention.

## Introduction

Non-trophoblastic tumors are the most common benign neoplasms of the placenta. They include chorioangioma, teratoma, leiomyoma and hepatocellular adenoma. Chorioangioma is the most common subtype [1]. Most commonly, chorioangiomas are small with negligible clinical significance [2]. Complications are associated with giant chorioangiomas (>4 cm) [3]. Increased vascularity of placental chorioangioma has been associated with an increased risk for fetal complications. Color Doppler imaging has a role in determining chorioangioma vascularity and therefore it is widely used in diagnosis and follow-up of placental chorioangiomas [4]. We hereby report a case of a spontaneous pregnancy complicated by placental chorioangioma at second trimester and the pregnancy outcome.

## Patient and observation

**Figure 1:**
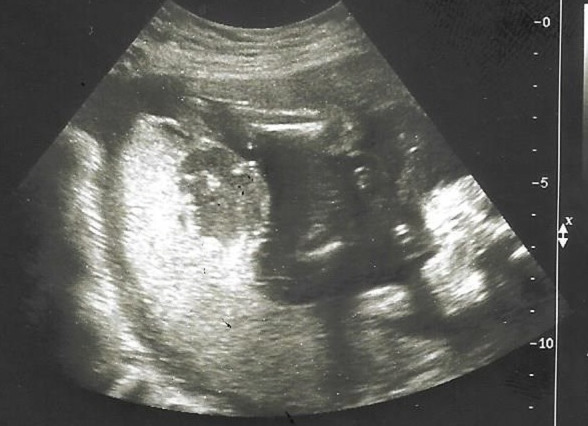
the placental chorioangioma on ultrasound at 21 weeks´ gestation

**Figure 2:**
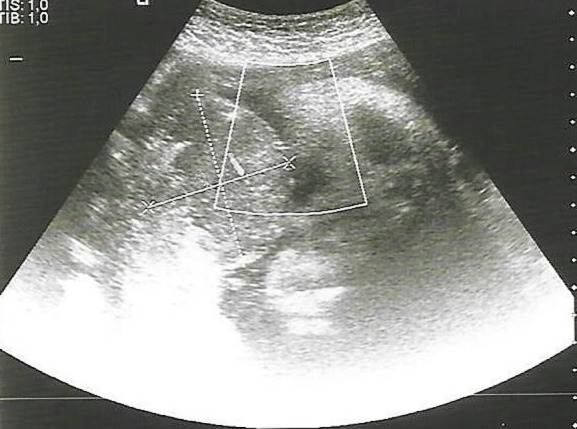
sonogram obtained at 34 weeks and 6 days´ gestation shows the chorioangioma measuring 6.27 x 5.38cm

**Figure 3:**
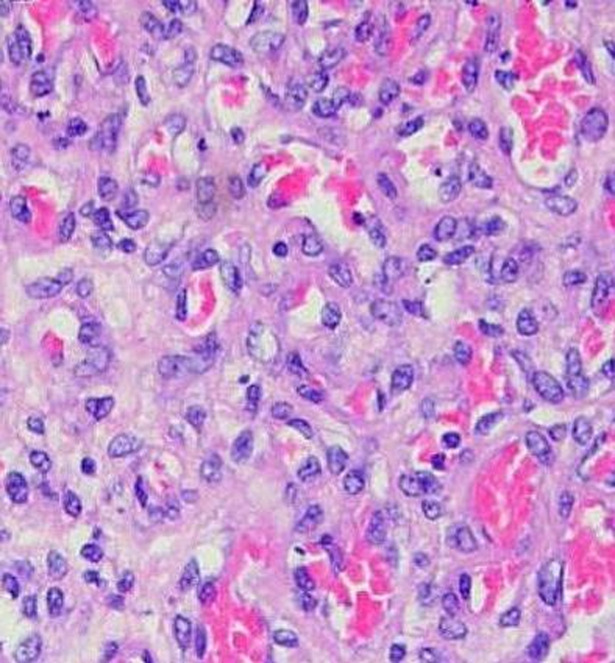
histologic feature of chorioangioma (hematoxylin-eosin stain)

A 29-year-old Caucasian primigravida woman with unremarkable medical history was noted on routine scan at 21 weeks of gestation to have a placental mass measuring 1.8 x 2.2 cm (Figure 1). A detailed ultrasound evaluation revealed a well circumscribed, hypoechoic lesion with increased vascularity, protruding into the amniotic cavity; thus a preliminary diagnosis of placental chorioangioma was made and close prenatal surveillance was scheduled. The fetus grew appropriately, middle cerebral artery peak systolic velocity as well as amniotic fluid index were within normal limits, as pregnancy advanced. At 34 weeks and 6 days of gestation, the mass in the placenta had increased in size to 6.27 x 5.38 cm (Figure 2). However, the patient presented to our emergency department complaining of reduced fetal movements at 37 weeks gestation. Sonographic evaluation did not suggest significant changes and cardiotocography was reassuring. Labor induction was recommended but the patient requested delivery by caesarean section. A small-for-gestational age (2650 g) but normal female neonate was delivered. Both mother and infant were discharged four days after delivery. Placenta was sent for pathologic examination which confirmed the diagnosis. Macroscopic histopathological analysis revealed a 17 x 14 x 3.5 cm placenta with a pale brown and homogenous tumor measuring 6 cm. Microscopically, the tumor represented an angiomatous-type chorioangioma consisting of numerous capillary-type vessels (Figure 3).

## Discussion

Chorioangioma is the most frequent non-trophoblastic benign tumor of the placenta, with an estimated incidence of 0.6% [5]. Maternal hypertension, gestational diabetes, twin pregnancy and female fetal sex increase placental chorioangioma incidence [6]. Most chorioangiomas are small, asymptomatic and generally have no clinical significance [2]. Giant chorioangiomas, greater than 4-5 cm in diameter, are rarely seen in obstetric practice, occurring in approximately 1 in 10000 pregnancies [7]. Ultrasound scanning can easily detect giant chorioangiomas, which are related to a number of serious adverse perinatal and fetal outcomes. Maternal complications include intrauterine fetal growth restriction, polyhydramnios and preterm labor, whereas fetal complications include fetal anemia, cardiomegaly, congestive fetal heart failure, nonimmune hydrops and fetal demise [3,8]. Sonographic features of placental chorioangioma include a solid hypoechoic, heterogeneous mass, which protrudes into the amniotic cavity on the fetal surface of the placenta. Doppler ultrasound assessment of chorioangioma is the key in characterizing the vascularity of the tumor, which confirms the diagnosis in contrast to other solid masses [9]. On magnetic resonance imaging (MRI), chorioangioma appear isointense to the placenta on T1-weighted images and have variable signal, usually being hyperintense to the placenta on T2-weighted images. In cases of large, very heterogeneous chorioangiomas, MRI can be helpful in differentiate chorioangiomas from other rare placental masses such as teratomas [10].

During histopathology examination, these tumors demonstrate angiomatous and cellular matrix with degenerative changes of calcification. In larger tumors, hyaline and myxoid degeneration is noticed [11]. Due to rarity of cases and diversity of their clinical features, no consensus has yet been established on management of placental non-trophoblastic tumors [1]. Close surveillance with regular sonograhic evaluation is necessary in order to intervene with proper therapeutic methods in a timely fashion. The management of giant placental chorioangioma depends on presence of fetal complications and gestational age. When complications associated with the tumor appear late in pregnancy, delivery should be considered. If complications develop earlier, delivery is not considered an option, due to prematurity [12]. Fetal transfusion for cases presenting with fetal anemia and amniodrainage in the presence of polyhydramnios, are two of the most common therapeutic procedures. Although, the results of these two interventions are favorable, they do not affect the underlying pathophysiology of internal arteriovenous shunts of the chorioangioma [3]. Definite prenatal treatment of placental chorioangioma is recommended for cases with life-threatening complications. Ultrasound-guided methods such as laser ablation, alcohol injection, microcoil embolization and ligation of blood vessels have been described in the literature, with fetoscopic laser ablation being the method of choice especially in chorioangiomas with small and superficial feeding vessels [1,13].

## Conclusion

In summary, diagnosis of placental chorioangioma by ultrasonography is only suggestive and the final diagnosis is established upon histological examination of the tumor. Our case proves that placental chorioangioma can be asymptomatic even when the size is greater than 4 cm. Moreover, MRI was not used antenatally to observe the characteristics of the placental tumor. Hence, transabdominal ultrasound is considered an acceptable method for regular surveillance of the mass during pregnancy. Our patient had a favorable outcome without any medical intervention and delivery was performed full-term. The neonate was not admitted to the neonatal unit, which demonstrates that fetal anemia was not developed despite the large size of the chorioangioma. Early prenatal diagnosis, close monitoring and appropriate intervention are considered critical in order to reduce fetal morbidity and mortality.
